# Vacuolated Blasts in the Bone Marrow of a Child with Rhabdomyosarcoma

**DOI:** 10.4274/tjh.galenos.2019.2019.0324

**Published:** 2020-02-20

**Authors:** Eda Ataseven, Dilek Ece, Nazan Özsan, Mehmet Kantar

**Affiliations:** 1 Ege University Faculty of Medicine, Department of Pediatric Hematology and Oncology, İzmir, Turkey; 2 Ege University Faculty of Medicine, Department of Pediatrics, Division of Pediatric Hematology and Oncology, İzmir, Turkey; 3 Ege University Faculty of Medicine, Department of Pathology, İzmir, Turkey; 4 Ege University Faculty of Medicine, Department of Pediatrics, Division of Pediatric Oncology, İzmir, Turkey

**Keywords:** Rhabdomyosarcoma, Vacuolated blasts, Bone marrow involvement

## To the Editor,

Rhabdomyosarcoma (RMS) is the most common soft tissue sarcoma in children. The most common locations are the head/neck region and genitourinary tract. Leukemic presentation of RMS with diffuse bone marrow involvement and unknown primary mass is very rare [1]. Most of the time it can be misdiagnosed as acute leukemia.

A 3-year-old female patient was admitted to the hospital with right arm pain, a limp while walking, and abdominal pain. From her medical history we learned that one month earlier she was diagnosed with a humerus fracture in the orthopedics clinic, and despite fixation her pain had gradually increased and disseminated. Magnetic resonance imaging of the humerus had revealed diffuse bone marrow edema and pathological lymph node enlargement in the axillary region. She was referred to our clinic with a suspicion of hematologic malignancy.

Upon physical examination, she had local swelling and pain in the right arm. There was no organomegaly or pathological lymph node enlargement on palpation. Laboratory examination revealed hemoglobin level of 10.3 g/dL, white blood cell count of 8.8x10^9^/L, and platelet count of 218x10^9^/L. Examination of the peripheral blood smear was normal. Biochemical test results were normal, except elevated lactate dehydrogenase and uric acid levels. Abdominal ultrasonography was normal. We performed bone marrow aspiration and biopsy with a suspicion of leukemia.

**Figure 1:**
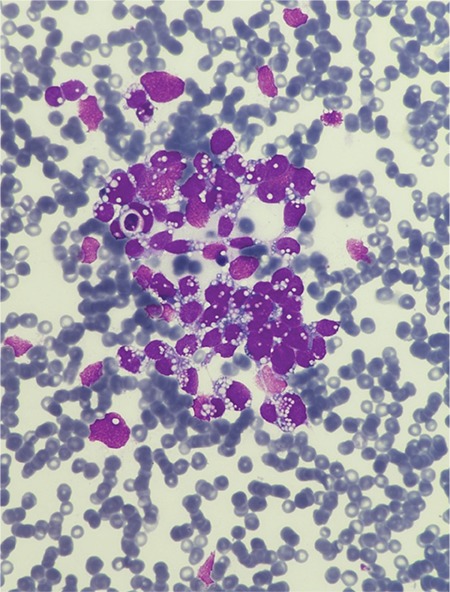
Bone marrow aspiration smear showed immature cells with disseminated intranuclear/intracytoplasmic vacuolization.

**Figure 2:**
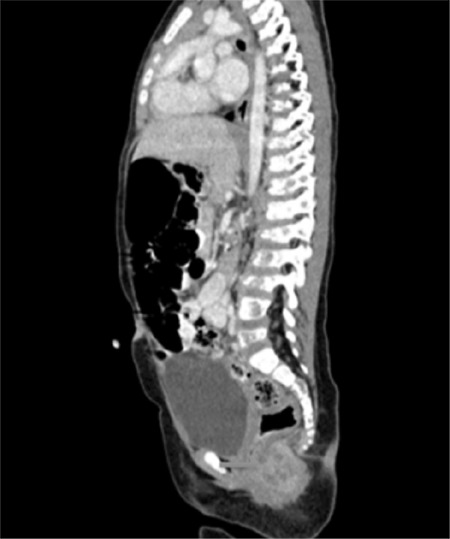
Abdominal CT showed a huge mass in pararectal fossa. CT: Computed tomography.

The bone marrow aspiration smear showed immature cells with disseminated intranuclear/intracytoplasmic vacuolization (Figure 1). In the bone marrow biopsy, diffuse blastic infiltration was noticed. Blastic cells were positive for myogenin and desmin staining. The diagnosis was RMS metastasis in the bone marrow. Work-up of the primary site of the disease was performed. Abdominal computed tomography showed a huge mass in the left pararectal fossa and multiple bone metastases (Figure 2).

RMS rarely involves the bone marrow. Blastic cells in RMS resemble lymphoid blasts and it can mimic acute leukemia or Burkitt’s lymphoma with marrow involvement, especially if there are prominent vacuolizations, as in our case [1,2]. In the literature, all patients presenting with bone marrow involvement were diagnosed with alveolar type RMS [3]. We could not distinguish the type of RMS in our patient. RMS should be kept in mind in the differential diagnosis of patients, especially if there are clustering blastic cells, multinucleated giant cells with deep blue cytoplasm, and prominent vacuolization [4].
